# Motor deficits are independent of axonopathy in an Alzheimer's disease mouse model of TgCRND8 mice

**DOI:** 10.18632/oncotarget.18429

**Published:** 2017-06-09

**Authors:** Qiuju Yuan, Jian Yang, Wutian Wu, Zhi-Xiu Lin

**Affiliations:** ^1^ School of Chinese Medicine, Faculty of Medicine, The Chinese University of Hong Kong, Hong Kong SAR, China; ^2^ Brain Research Centre, Faculty of Medicine, The Chinese University of Hong Kong, Hong Kong SAR, China; ^3^ School of Biomedical Sciences, Li Ka Shing Faculty of Medicine, The University of Hong Kong, Hong Kong SAR, China; ^4^ State Key Laboratory of Brain and Cognitive Sciences, Li Ka Shing Faculty of Medicine, The University of Hong Kong, Hong Kong SAR, China; ^5^ Research Center of Reproduction, Development and Growth, Li Ka Shing Faculty of Medicine, The University of Hong Kong, Hong Kong SAR, China; ^6^ GHM Institute of CNS regeneration, Jinan University, Guangzhou, China

**Keywords:** axonal transport, dystrophic axon swellings, corticospinal tract, motor deficits

## Abstract

There have been an increasing number of reports of non-cognitive symptoms in Alzheimer's disease (AD). Some symptoms are associated with the loss of motor functions, e.g. gait disturbances, disturbed activity level and balance. Consistent with clinical findings, several AD mouse models harboring amyloid pathology develop motor impairment. Although the factors that contribute to the motor deficits have not yet been determined, it has been suggested that axonopathy is one of the key factors that may contribute to this particular feature of the disease. Our previous study found that TgCRND8 mice exhibited profound motor deficits as early as 3 months old. In this study, we explored the possible factors that may be related to motor deficits in TgCRND8 mice. Results from silver, neurofilament and amyloid precursor protein (APP) staining revealed no axonopathy occurred in the brain and spinal cord of TgCRND8 mice at the age of 3 months. Anterograde labeling of corticospinal tract of spinal cord and electronic microscopy (EM) analysis showed that no axonopathy occurred in TgCRND8 mice at the age of 3 months. According to these results, it could be concluded that no axonal alterations were evident in the TgCRND8 mice when motor deficits was overt. Thus, axonopathy may play a less prominent role in motor deficits in AD. These results suggest that mechanisms by which motor function undergo impairment in AD need to be further studied.

## INTRODUCTION

Alzheimer's disease (AD) is the most common form of dementia in the elderly population worldwide [1]. Its main clinical symptoms include memory impairment and global cognitive deficits that can lead to dementia with the disease progression [1–3]. Although the exact etiology of AD is not fully understood so far, substantial evidence indicates that amyloid-β peptide (Aβ), derived from sequential cleavage of amyloid-β protein precursor (APP) by β- and γ-secretases, plays a central role in the pathogenesis of AD [2, 4]. Apart from memory impairment and cognitive deficits, there have been increasing reports that non-cognitive symptoms, especially loss of motor functions, such as gait disturbances, disturbed activity level and balance, are associated with incident AD [5–8]. Compared with those who are cognitively intact, lower level of motor functions may be more pronounced in older persons with cognitive impairment [9–12]. Furthermore, loss of motor functions can precede cognitive impairment by several years [9, 11–16]. Both a lower level and more rapid rate of motor decline in cognitively intact individuals predict the subsequent development of mild cognition impairment and AD.

Consistent with clinical studies, several AD mouse models harboring amyloid pathology with the development of motor impairment and spinal pathology have been developed in 5XFAD mice [17], APP/PS1ki mice [18, 19] and Tg2576 mice [20]. All these mice show an abnormal hindlimb extension reflex when suspended by tails, i.e., retracting both forelimbs and hindlimbs simultaneously [17–20]. This pattern is completely absent in control mice [17–20].

TgCRND8 mouse expresses a transgene incorporating both the Indiana mutation (V717F) and the Swedish mutation (K670N/M671L) in the human amyloid-β protein precursor (APP) gene [21]. TgCRND8 mouse is well-known for reproducing important features of AD including amyloid plaques, hyperphosphorylation of Tau, and cognitive deficits [22]. Interestingly, we have observed profound motor deficits of the TgCRND8 mice as early as 2–3 months old [23] when few, if any, AD Aβ neuropathologies can be observed in either brain or spinal cord of TgCRND8 mice [24]. This is consistent with the clinical observation that motor deficits may be preclinical phenotype of AD. Recently, many researchers prompt that the motor deficits may be caused by a disruption of axonal transport, as demonstrated by axonopathy including axonal swellings and spheroids [17–19]. Therefore, it may be possible that axonopathy occur in TgCRND8 mice at the age of 3 months. However, there is no evidence to support this hypothesis so far. The purpose of this study was thus to investigate whether TgCRND8 mice undergo axonopathy that is related to motor deficits. One method of studying changes in axons is using antibodies against a variety of axonal markers, for example neurofilaments (NFs). NFs are exclusively expressed in neurons where they are essential for maintaining cell shape and facilitating intracellular transport. Abnormal accumulation of NF together with disease-specific protein aggregation has been identified in a number of neurodegenerative disorders, including amyotrophic lateral sclerosis [25], AD [19, 26] and Parkinson's disease [27]. Hence, it presents an ideal method to study axonopathy and has been used to detect axonopathy in several AD mice models [28]. There are also alternative staining methods for detecting axonopathy, such as APP immunostaining, Bielschowsky silver staining [29], and anterograde labeling of axons [30]. All these methods were applied in this study to examine whether TgCRND8 mice may undergo axonopathy or not.

## RESULTS

### Early onset of motor deficits in TgCRND8 mice at the age of 3 months

**Figure 1:**
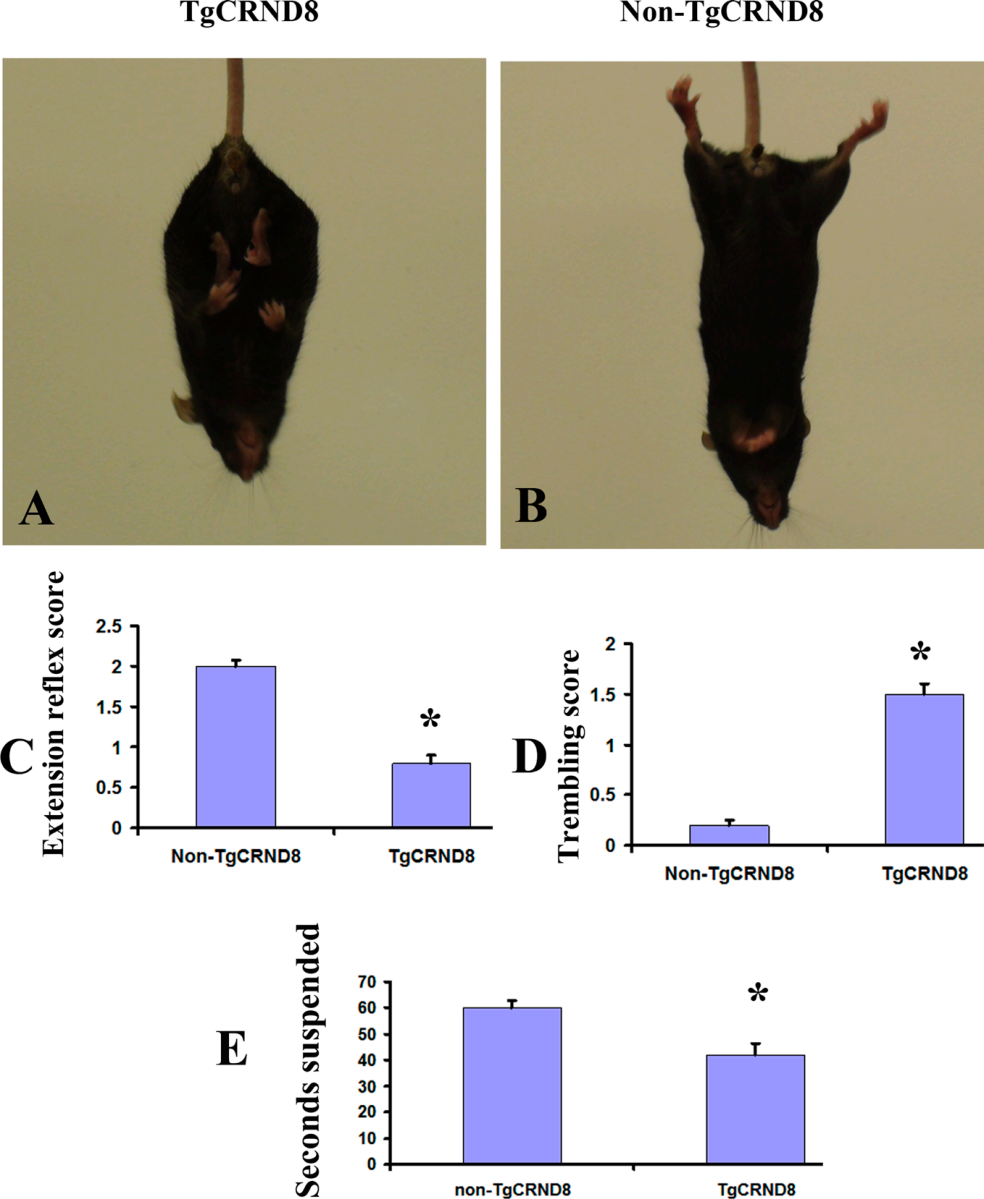
Abnormal motor function phenotypes in TgCRND8 mice at the age of 3 months (**A**) and (**B**) Abnormal limb flexion in TgCRND8 mice at the age of 3 months. (A) a TgCRND8 mouse fails to spread its hindlimbs when suspended by the tail. (B) an age-matched non-TgCRND8 mouse spreads its hindlimbs when suspended by the tail. (**C**) Quantification of scored hindlimb extension reflex of the TgCRND8 and their non-TgCRND8 mice. (**D**) Quantification of scored body trembling and hindlimb tremor of TgCRND8 and age-matched non-TgCRND8 mice at the age of 3 months. (**E**) TgCRND8 mice reduced motor abilities in a balance beam task compared to age-matched non-TgCRND8 mice.

We observed that TgCRND8 mice displayed abnormal motor function phenotypes as early as 3 months of age. While being suspended by the tail, the TgCRND8 mice held and retracted the hindlimbs in the inward direction (Figure 1A and 1C); in starkly contrast to the stretching of the hindlimbs in the outward direction in non-TgCRND8 mice (Figure 1B and 1C). The TgCRND8 mice also displayed severe body trembling and hindlimb tremors when suspended by the tail (Figure 1D) (*p* < 0.05). To study the sensorimotor skills in more detail, the mice were then analysed with the traversing beam challenge. The TgCRND8 mice showed reduced motor abilities when compared with their age-matched non-TgCRND8 controls (Figure 1E) (*p* < 0.05).

### Absence of NF-200 positive axonopathy in TgCRND8 mice at the age of 3 months

**Figure 2:**
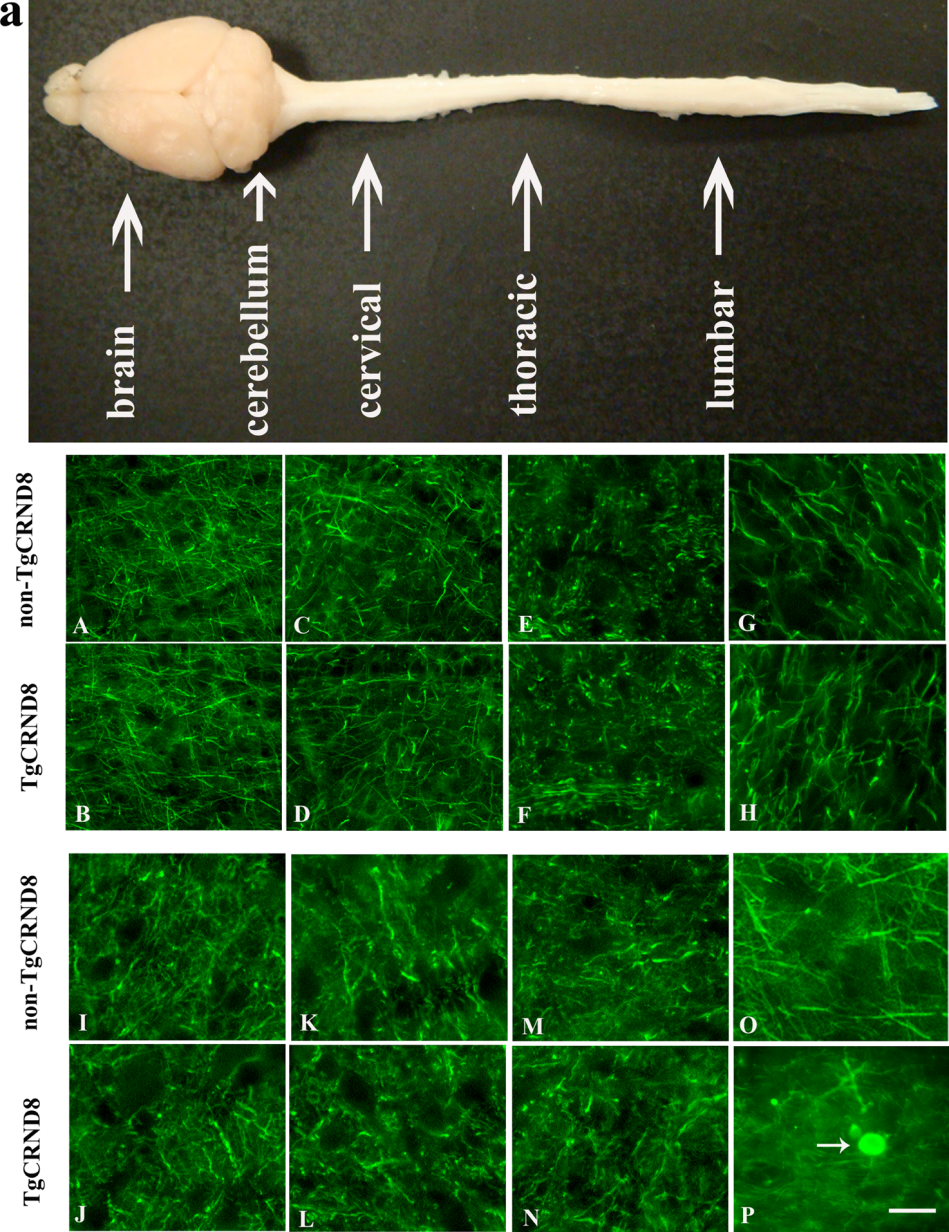
NF200 immunohistochemical staining indicated no axonopathy in the brain and spinal cord of TgCRND8 mice at the age of 3 months compared to age-matched non-TgCRND8 mice a: a gross picture of mouse central nervous system including brain, spinal cord at cervical, thoracic and lumbar level. (**A**–**H**) no NF-positive axonal swellings and spheroids were observed in different brain regions including cortex (B), hippocampus (D), cerebellum (F), pons (H) of TgCRND8 mice, which were comparable to that in non-TgCRND8 mice (A, C, E and G, respectively). (**J**–**M**) no NF-positive axonal swellings and spheroids were detected in the different spinal cord segments of cervical (J), thoracic (L), lumbar (**N**) in the TgCRND8 mice, which were comparable to that in the non-TgCRDN8 mice (**I**, K and M, respectively). (**O**–**P**) NF-positive axonal swellings and spheroids were observed in the spinal cord of the TgCRND8 at the age of 9 months (arrow in P) but not in age-matched non-TgCRND8 mice (O). Scale bars = 50 μm.

We analyzed different regions of brain in TgCRND8 mice and non-TgCRND8 control mice using antibodies against 200-kDa NF (NF200). TgCRND8 mice of 3 months of age did not show any evidence of axonopathy revealed by NF-positive axonal swellings and spheroids in cortex (Figure 2A), hippocampus (Figure 2C), cerebellum (Figure 2E), and pons (Figure 2G), which was comparable to non-TgCRND8 mice (Figure 2B, 2D, 2F and 2H, respectively). We also analyzed different segments of spinal cord. Either TgCRND8 mice or non-TgCRND8 mice of 3 months of age did not show axonopathy in the cervical (Figure 2I and Figure 2J, respectively), thoracic (Figure 2K and Figure 2L, respectively) and lumbar (Figure 2M and Figure 2N, respectively). However, a small number of NF-positive axonal swellings and spheroids were observed in the spinal cord of TgCRND8 mice at the age of 9 months (arrow in Figure 2P) while its non-TgCRND8 control mice did not show axonopathy (Figure 2O).

### Absence of APP positive axonopathy in TgCRND8 mice at the age of 3 months

**Figure 3:**
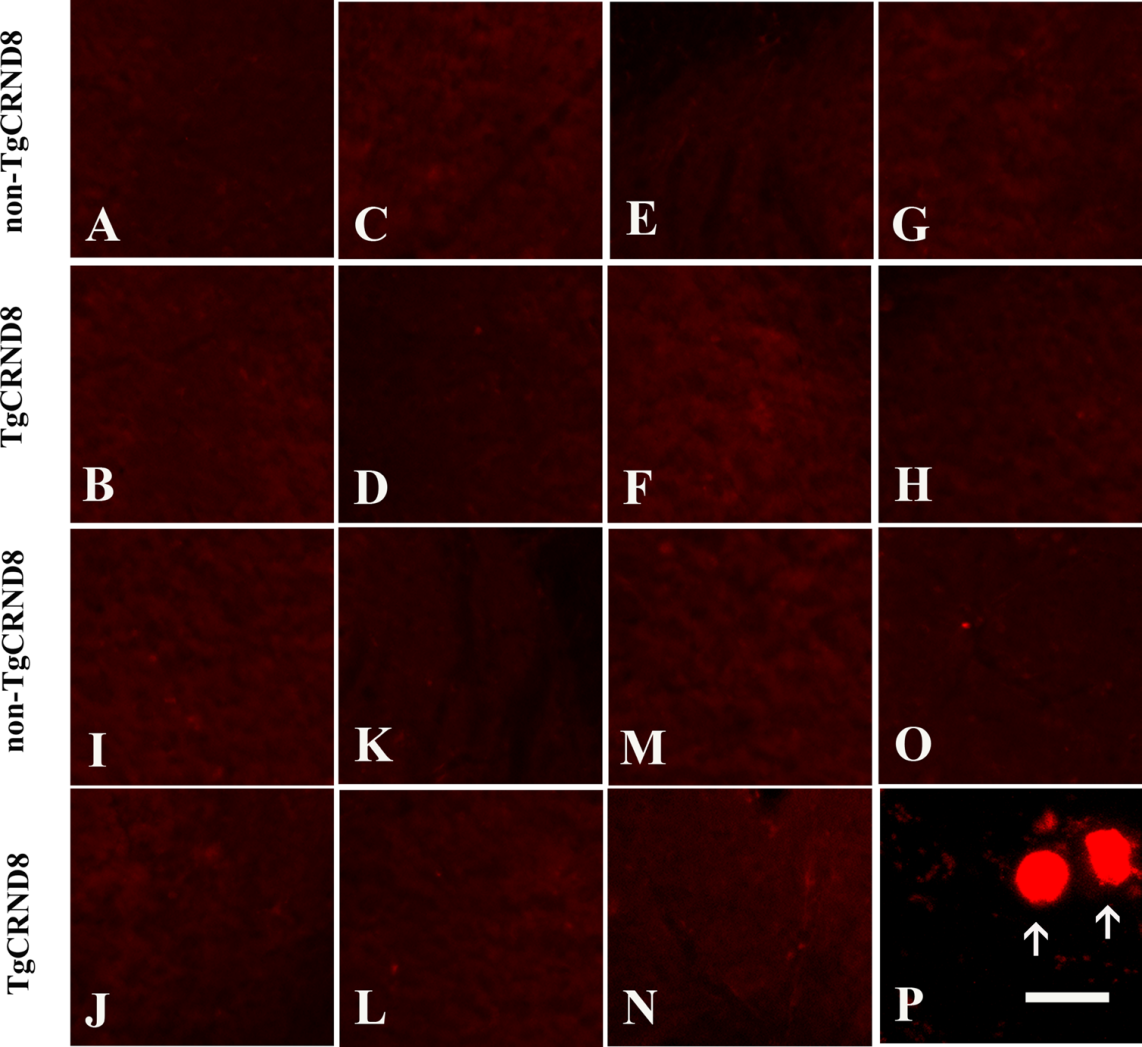
APP immunohistochemical staining indicated no axonopathy in the brain and spinal cord of TgCRND8 mice at the age of 3 months compared to age-matched non-TgCRND8 mice (**A**–**H**) no NF-positive axonal swellings and spheroids were observed in different brain regions including cortex (B), hippocampus (D), cerebellum (F), pons (H) of TgCRND8 mice, which were comparable to that in non-TgCRND8 mice (A, C, E and G, respectively). (**J**–**M**) no NF-positive axonal swellings and spheroids were detected in the different spinal cord segments of cervical (J), thoracic (L), lumbar (**N**) in the TgCRND8 mice, which were comparable to that in the non-TgCRDN8 mice (**I**, K and M, respectively). (**O**–**P**) NF-positive axonal swellings and spheroids were observed in the spinal cord of the TgCRND8 at the age of 9 months (arrows in P) but not in age-matched non-TgCRND8 mice (O). Scale bars = 10 μm.

We further analyzed different regions of brain in TgCRND8 mice and non-TgCRND8 control mice using antibodies against APP. TgCRND8 mice of 3 months of age did not show any evidence of axonopathy revealed by APP-positive axonal swellings and spheroids in cortex (Figure 3A), hippocampus (Figure 3C), cerebellum (Figure 3E), and pons (Figure 3G), which was comparable to non-TgCRND8 mice (Figure 3B, 3D, 3F and 3H, respectively). We also analyzed different segments of spinal cord. Either TgCRND8 mice or non-TgCRND8 mice of 3 months of age did not show axonopathy in the cervical (Figure 3I and Figure 3J, respectively), thoracic (Figure 3K and Figure 3L, respectively) and lumbar (Figure 3M and Figure 3N, respectively). However, a small number of APP-positive axonal swellings and spheroids were observed in the spinal cord of TgCRND8 mice at the age of 9 months (arrow in Figure 3P) while its non-TgCRND8 control mice did not show axonopathy (Figure 3O).

### Absence of axonopathy by silver staining in TgCRND8 mice at the age of 3 months

**Figure 4:**
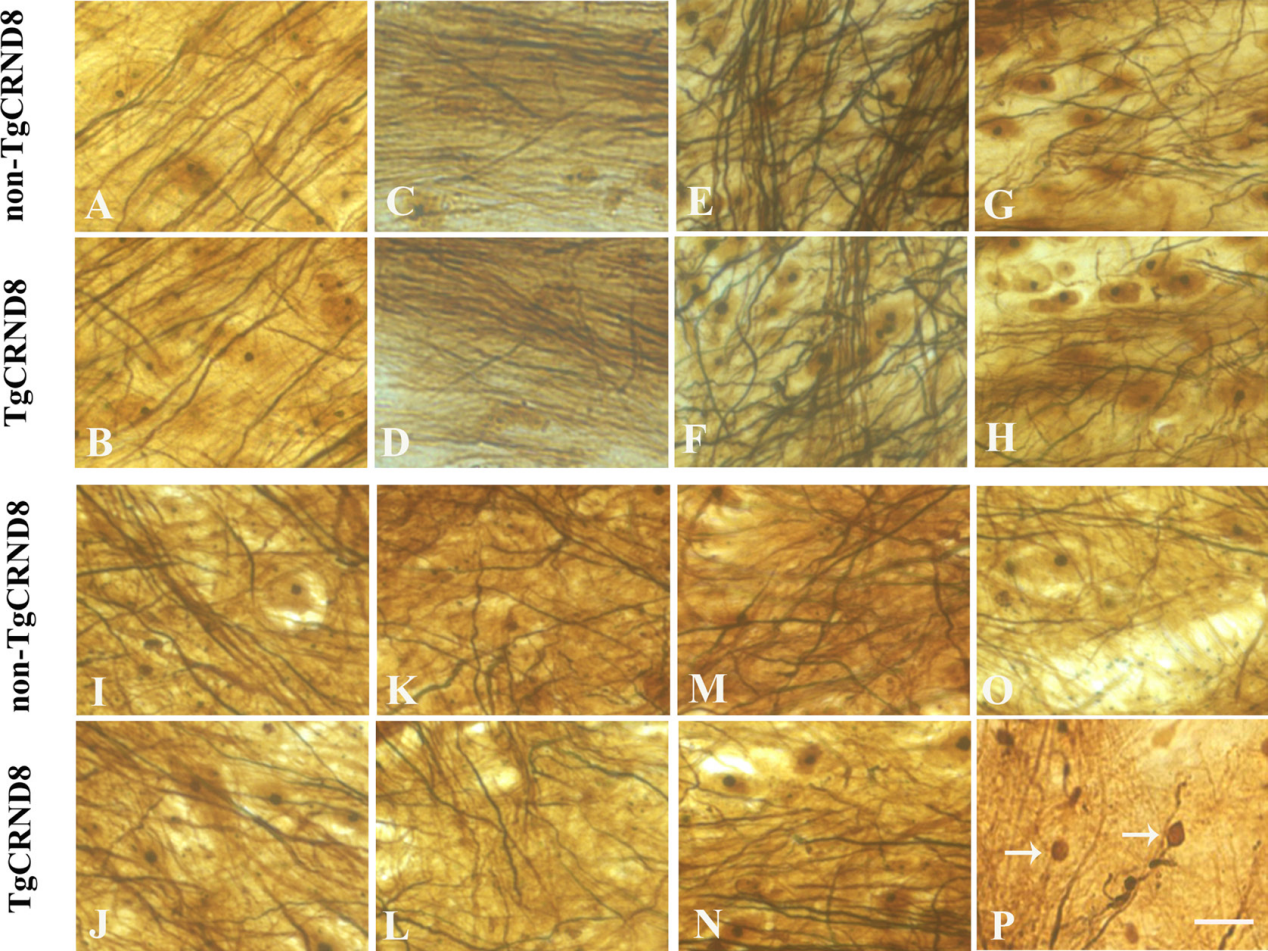
Representative Bielschowsky silver staining demonstrated the absence of axonopathy in various brain regions of TgCRND8 mice at the age of 3 months, including cortex (**B**), hippocampus (**D**), cerebellum (**F**), pons (**H**) and various spinal cord segments of cervical (**J**), thoracic (**L**) and lumbar (**N**), which was comparable to that in the age-matched non-TgCRND8 mice (**A**, **C**, **E**, **G**, **I**, **K** and **M**, respectively). Bielschowsky silver staining demonstrated axonopathy in the spinal cord of TgCRND8 at the age of 9 months (arrows in **P**) but not in age-matched non-TgCRND8 mice (**O**). Scale bars = 50 μm.

Furthermore alternative staining with the Bielschowsky silver procedure revealed similar pattern with NF immunostaining. Either TgCRND8 or non-TgCRND8 littermate control mice at the age of 3 months did not show axonopathy in cortex (Figure 4A and 4B, respectively), hippocampus (Figure 4C and 4D, respectively), cerebellum (Figure 4E and 4F, respectively), and pons (Figure 4G and 4H, respectively). Similarly, either TgCRND8 mice or non-TgCRND8 mice of 3 months of age did not show axonopathy in the cervical (Figure 4I and 4J, respectively), thoracic (Figure 4K and 4L, respectively) and lumbar (Figure 4M and Figure 4N, respectively). However, TgCRND8 (arrows in Figure 4P) but not non-TgCRND8 (Figure 4O) mice at the age of 9 months exhibited axon spheroids in the spinal cord.

### Absence of axonopathy in the corticospinal tract of spinal cord of TgCRND8 mice at the age of 3 months

**Figure 5:**
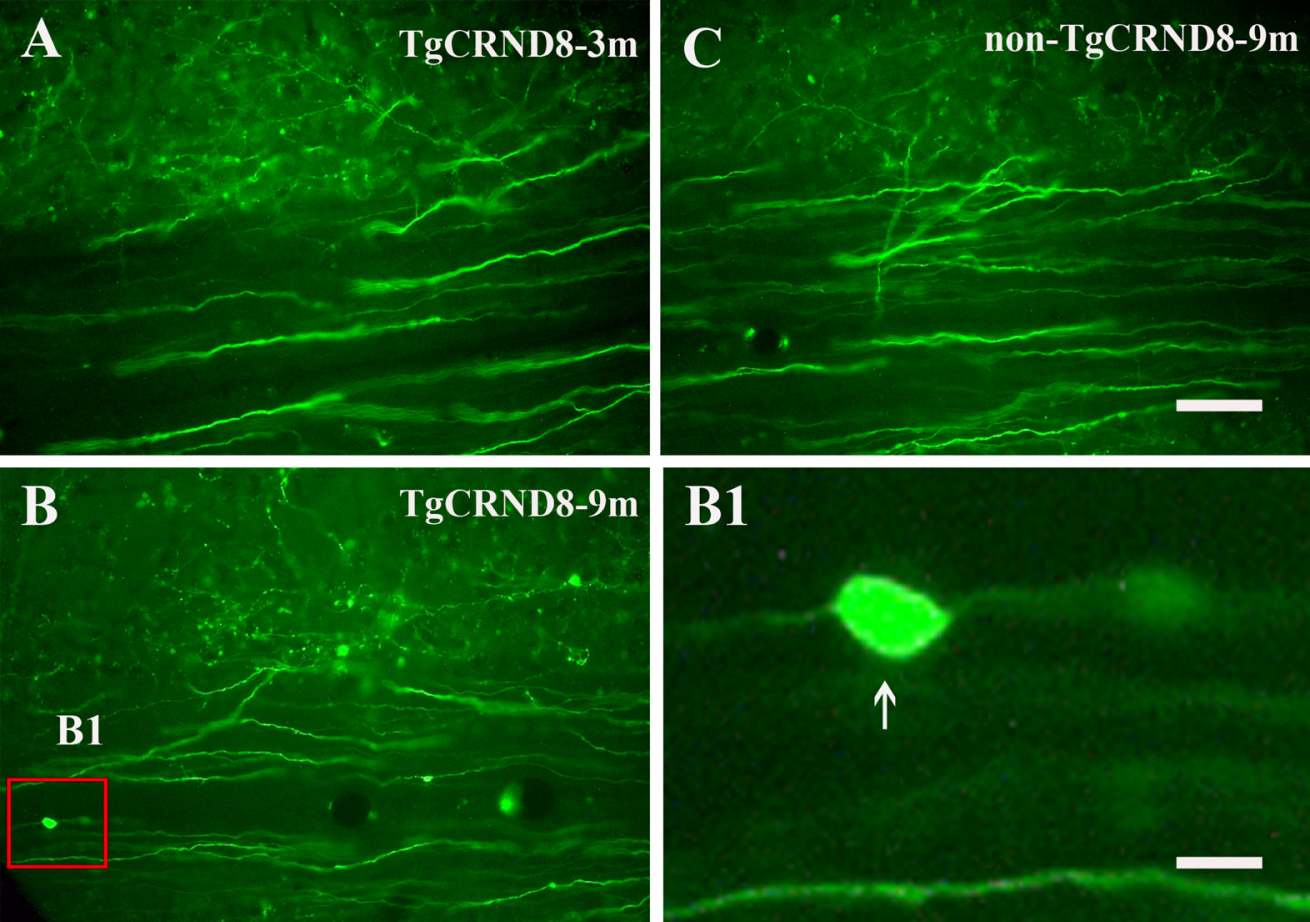
Representative anterograde labeling demonstrated the absence of axonal swellings and spheroids in the corticospinal tract of the spinal cord in TgCRND8 mice at the age of 3 months (**A**). Axonal swellings and spheroids were observed in the spinal cord of the TgCRND8 at the age of 9 months (inset in (**B**) and arrow in B1) but not in age-matched non-TgCRND8 mice (**C**). **B1** is the magnification of the inset in B. Scale bar in C = 100 μm. Scale bar in B1= 15 μm.

Anterograde labeling of corticospinal tract revealed that corticospinal tract of spinal cord of TgCRND8 mice did not undergo axonal pathology at the age of 3 months (Figure 5A). However, TgCRND8 (inset in Figure 5B and arrow in 5B1) but not non-TgCRND8 (Figure 5C) mice at the age of 9 months exhibited axon spheroids in the spinal cord.

### Absence of axonopathy in the spinal cord of TgCRND8 mice by electronic microscopy (EM) at the age of 3 months

**Figure 6:**
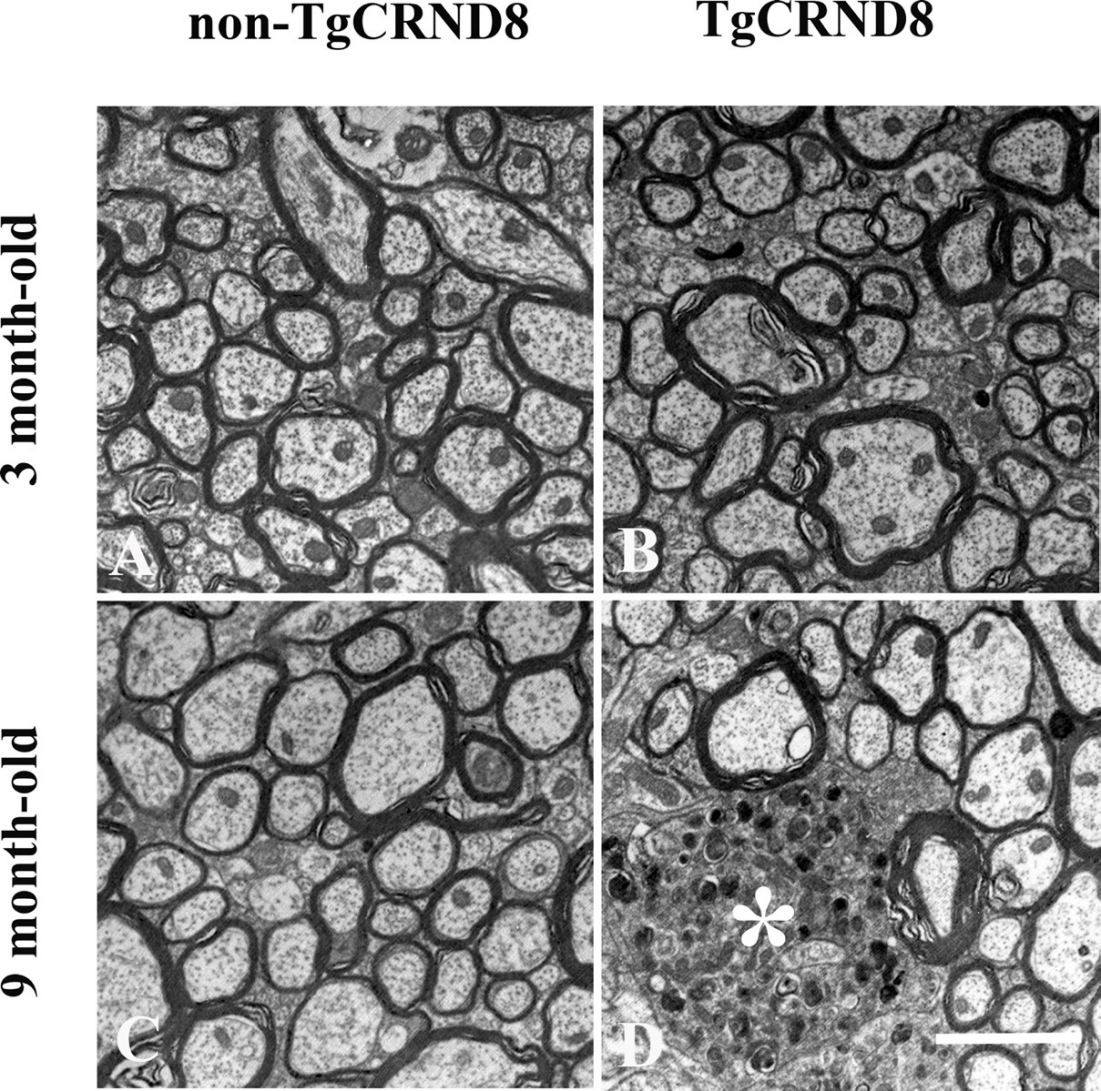
Ultrastructural analysis of axon status of the spinal cord demonstrated the absence of axonopathy in the spinal cord in either TgCRND8 mice (**B**) or non-TgCRND8 mice (**A**) at the age of 3 months. Dilated axons were observed in the spinal cord of the TgCRND8 mice at the age of 9 months (^*^ in **D**) but not in age-matched non-TgCRND8 mice (**C**). Scale bar = 6 μm.

Spinal cord cervical segments from TgCRND8 and non-TgCRND8 mice at the age of 3 and 9 months were analyzed by electron microscopy (Figure 6). The ultrastructure analysis revealed that the spinal cord of either TgCRND8 (Figure 6B) or non-TgCRND8 (Figure 6A) mice did not undergo axonal pathology at the age of 3 months. However, TgCRND8 (^*^ in Figure 6D) but not non-TgCRND8 (Figure 6C) mice at the age of 9 months exhibited dilated axons in the spinal cord. The dilated axons were filled with electron dense material and numerous vesicular bodies and absent myelin sheath (^*^ in Figure 6D).

### Absence of Aβ plaques in spinal cord of TgCRND8 mice at the age of 3 months

**Figure 7:**
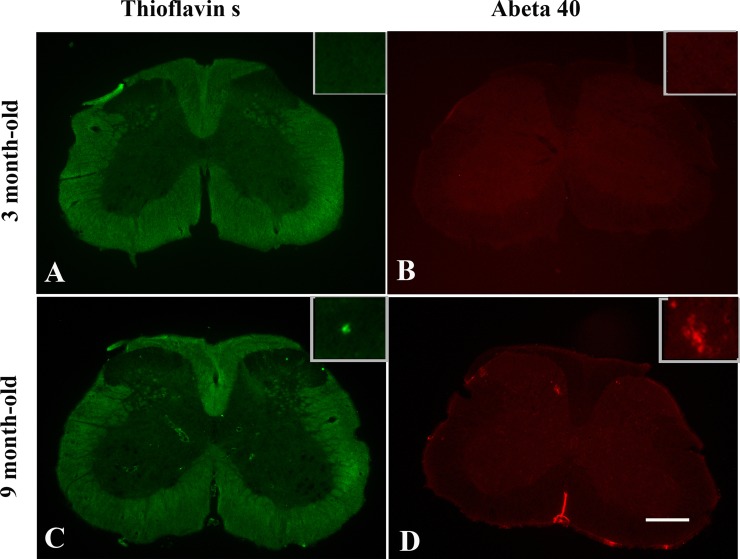
Age-dependent senile plaque pathology in the spinal cord of TgCRND8 mice Both Thioflavin S staining (for dense-core plaques) and immunohistochemical staining for Aβ demonstrated Aβ plaques presented in the spinal cord at the age of 9 months (**C** and **D**, respectively). No Aβ plaques were detected in TgCRND8 mice at the age of 3 months (**A** and **B**, respectively). Scale bar = 150 μm.

Thioflavin S staining (for dense-core plaques) and immunohistochemical staining for Aβ were used to analyze plaque pathology in spinal cord of TgCRND8 mice. At 3 months of age, no amyloid plaques were detected in spinal cord of TgCRND8 mice (Figure 7A and 7B), whereas some amyloid plaques were detected in spinal cord of TgCRND8 mice at the age of 9 months (Figure 7C and 7D).

### Absence of motoneuron or motor axon loss in TgCRND8 mice at the age of 3 months

**Figure 8:**
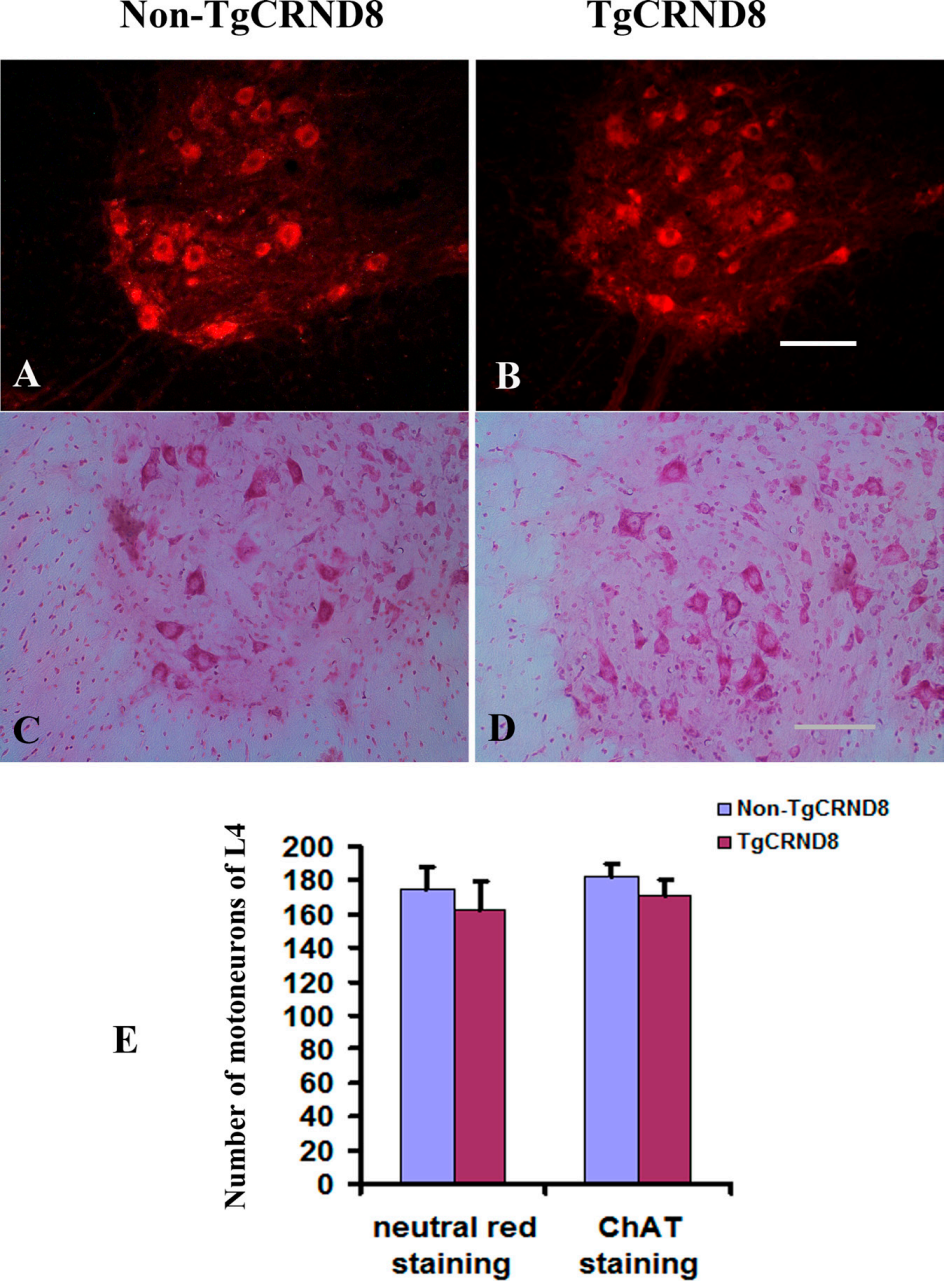
Absence of motoneuron loss in the lumbar cord of TgCRND8 mice at the age of 3 months (**A**) and (**B**) Representative photomicrographs showed neutral red-stained lumbar 4 (L4) spinal cord of non-transgenic control (A) and TgCRND8 mice (B) at the age of 3 months. (**C**) and (**D**) Representative photomicrographs showed anti-ChAT-stained lumbar L4 spinal cord of non-transgenic control (C) and TgCRND8 mice (D) at the age of 3 months. (**E**) Quantification of the numbers of neutral red stained motoneurons and anti-ChAT-stained cells in the ventral horn of the L4 of non-transgenic control and TgCRND8 mice at the age of 3 months. Scale bar in B and D = 200 μm.

**Figure 9:**
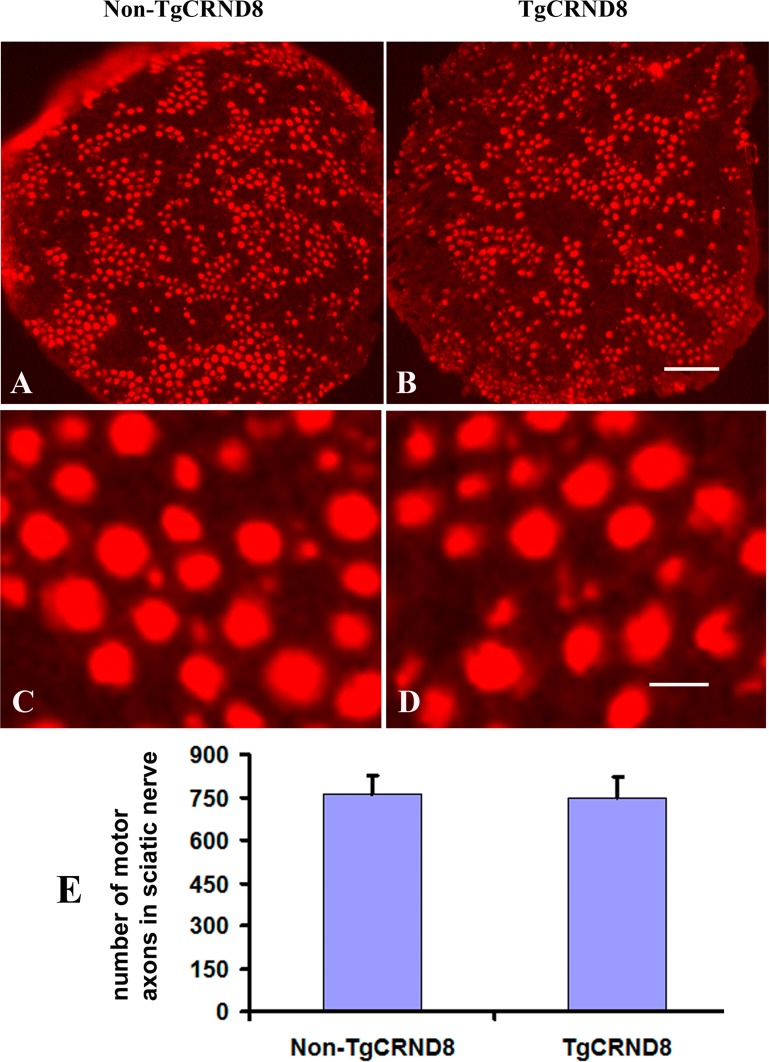
Absence of motor axon loss in the sciatic nerve of TgCRND8 mice (**A**) and (**B**) Representative photomicrographs showed anti-ChAT-stained sciatic nerve of non-transgenic control (A) and TgCRND8 mice (B) at the age of 3 months. (**C**) and (**D**) are the higher magnifications of A and B, respectively. (**E**) Quantification of the numbers of anti-ChAT-stained motor axons in the sciatic nerve of non-transgenic control and TgCRND8 mice at the age of 3 months. Scale bar in B = 200 μm. Scale bar in D = 15 μm.

Neutral red staining showed that the numbers of neutral red-stained cells in the ventral horn of the TgCRND8 mice at the age of 3 months (Figure 8B and 8E), which were comparable to that in the non-TgCRND8 control mice (Figure 8A and 8E). The number of anti-ChAT-positive neurons in the lumbar cord of the TgCRND8 mice (Figure 8D and 8E) were also similar to that of their controls (Figure 8C and 8E). Consistent with these findings, the number of anti-ChAT-positive motor axons in the sciatic nerve of the TgCRND8 mice (Figure 9A, 9C and 9E) was also similar to that of their controls (Figure 9B, 9D and 9E), indicating spinal motoneurons in TgCRND8 might not be affected in TgCRND8 mice at the age of 3 months.

## DISCUSSION

Previous studies in AD mice mainly focused on functions and dysfunctions of the AD brain, with less attention to the spinal cord. Since human AD patients often show motor deficits [31], increasing number of studies have paid attention to neuropathology in the spinal cord in various AD transgenic mice [17–20]. It has been suggested that Aβ plaque pathology in spinal cord may contribute to the motor deficit in AD mice. Indeed, the motor deficits are coincident with the occurrence of Aβ plaque in spinal cord in APP/PS1 ki [18, 19] and 5XFAD [17] mice. In our studies, we found that TgCRND8 mice showed a severe defect in the hindlimb extension reflex test and abnormal body trembling and hindlimb tremors when suspended by the tail. These abnormalities were overt at 3 months of age. Our histological analyses showed that TgCRND8 exhibited region-specific Aβ plaque pathogenesis in corticospinal tract pathway of the spinal cord [23], the major pathway for motor function. However, we could not correlate the motor phenotype with the region-specific Aβ deposition in corticospinal tract pathway of the spinal cord because this spinal cord plaque pathology cannot be detected before the age of 9 month in TgCRND8 mice, much older than the age when the motor deficits present. Recently, many researchers prompt that the early behavior deficit may be caused by a disruption of axonal transport, as demonstrated by axonopathy including axonal swellings and spheroids in spinal cord [17–19], because they have noted that the APP/PS1ki or 5XFAD mice show a large number of characteristic axonal swellings, spheroids, axonal ovoids in the spinal cord when motor deficits present. We also observed axonopathy in the spinal cord of TgCRND8 mice. However, we could not attribute the motor deficits to the axonopathy in the spinal cord of TgCRND8 mice because this axonopathy cannot be detected before the age of 9 month in TgCRND8 mice, much older than the age when the motor deficits present. We failed to detect any axonopathy in either spinal cord or brain of TgCRND8 mice at the age of 3 months. According to these findings that no axonal alterations were evident in the TgCRND8 mice when motor deficits were overt, it might be concluded that axonopathy may play a less prominent role in motor deficits in AD.

Seo et al. (2010) reported that Tg2576 mice, another popular AD mice, also presented motor function deficits. They speculated that these deficits might be associated with severe spinal motoneuron and its axon loss, since they found that Tg2576 mice showed severe motoneuron loss in the spinal cord and axonal degeneration of the sciatic nerves. In our study, we investigated whether motor deficits were resulted from severe motoneuron loss in the spinal cord and axonal degeneration of the sciatic nerves in TgCRND8 mice. Unfortunately, we did not find motoneuron and motor axon loss in TgCRND8 mice at any ages examined as they did. Obviously, the motor deficits in TgCRND8 could not be attributed to spinal motoneuron and its axon loss as in Tg2576 mice because they appeared normal when compared with those in non-transgenic controls.

Motor functions derive from the coordinated activities of varied motor control systems located throughout the brain and spinal cord, which extend via the peripheral nervous system to musculoskeletal structures [32, 33]. Motor control systems that regulate the initiation, planning and execution of motor performances are located in multiple interconnected cortical and subcortical motor regions [34, 35–38]. Descending white matter tracts provide the means for these supraspinal motor systems to influence spinal motor systems that directly control muscle, the final effector of all movement [39–43].

In contrast to strength testing that depends mostly on motor units and muscle function, motor performances, including gait and balance, reflect the functional integrity of widely distributed cortical and subcortical motor-related brain regions. Motor impairment may derive from damage to the integrity of the gray matter of motor-related brain regions and/or from damage of connectivity of whiter matter of motor-related brain regions.

A recent study has shown early alterations in brain functional connectivity (FC) in ArcAβ transgenic mice - another AD mouse model [44]. By using resting-state functional MRI (fMRI) methods, they assessed the FC in mice across their life-span with a cross-sectional design, and found that the mice showed compromised development of FC during the first month of postnatal life compared with wild-type animals, resulting in functional impairments that affect in particular the sensory-motor cortex, an important motor-related brain region, already in preplaque stage. Accordingly, we speculate that FC impairment may occur in the sensory-motor cortex at the age of 3 months in the TgCRND8 mice and the abnormality may result in motor deficits in gait and balance. In our further study, we will use resting-state fMRI methods to analyze the changes in FC in TgCRND8 mice.

## MATERIALS AND METHODS

### Transgenic mice

TgCRND8 mice express a transgene incorporating both the Indiana mutation (V717F) and the Swedish mutations (K670N ∕ M671L) in the human amyloid-beta protein precursor (APP) gene. The mice were kept on a C57BL6/J genetic background. In this study, TgCRND8 mice and their non-TgCRND8 littermates at the ages of 3 and 9 months were used (*n* = 5 in each group). All animals were handled in accordance with the animal care guidelines of the Committee on the Use of Live Animals for Teaching and Research of the University of Hong Kong.

### Hindlimb extension reflexes

Hindlimb extension reflexes were evaluated according to the procedure and scoring system described by Seo et al. [20]. Briefly, mice were suspended by the tail, and the degree of motor deficit was scored on a 0 to 2 scale: a normal extension reflex in both hindlimbs was scored as 2; imbalanced extension in the hindlimbs as 1.5; extension reflex in only one hindlimb as 1.0; the absence of any hindlimb extension as 0.5; and total paralysis as 0.

### Balance beam

To evaluate balance and general motor abilities, we followed the method described previously [35]. Briefly, a 1 cm wide wooden beam was attached to two support columns 44 cm above a padded surface (to protect against fall injuries). At either end of the 51 cm long beam, a 14 cm × 10 cm wooden platform was attached. Animals were placed at the center of the beam and released. The time (in seconds) for mice to fall from the beam was recorded. The shorter the time that the mice remained in the beam, the severer the motor deficits including impairment of balance and general motor abilities the mice displayed. If a mouse was able to remain on the beam for the whole duration of the 60-s trial or traverse beam to one of the attached platforms, the maximum time of 60 s was recorded. Mice were tested three times and the results were averaged.

### Body trembling and hindlimb tremor assessments

Body trembling and hindlimb tremors were evaluated according to the procedure and scoring system described by Seo et al. [20]. Briefly, mice were suspended by tails, and the degree of body trembling and hindlimb tremors was scored using a 0–4 rating scale: 0, normal in both hindlimbs and body; 1, weak tremors (1–5 frequency/ 10 sec) of the hindlimbs and body; 2, intermediate tremors (6–10 frequency/10 sec) of the hindlimbs and body; 3, severe tremors (11–15 frequency/10 sec) of the hindlimbs and body; very severe (16 and higher frequency/10 sec) of the hindlimbs and body.

### Anterograde labeling of the corticospinal tract in TgCRND8 mice

Since the corticospinal tract is the major pathway for motor function, we examined whether corticospinal tract of spinal cord of TgCRND8 mice undergoes axonopathy. Corticospinal tract labeling was performed in 2 and 7 month-old TgCRND8 mice (*n* = 5). As described in our previous study, the mice underwent anesthesia with ketamine (80 mg/kg) and xylazine (8 mg/kg). Craniotomy on right side was created with a surgical drill and 10% fluorescein conjugated dextran amine (dextran, fluorescein, 10,000. M.W. D1820, Molecular Probes Inc, Eugene, OR, USA) was injected into the sensorimotor cortex on the right side [32]. Five injections of 0.2 μl were performed at a depth of 0.5 mm from the cortical surface. The injections were placed between 1 mm anterior and 2 mm posterior to the Bregma and 2 to 3 mm from the midline (sagittal suture). Animals were killed with pentobarbital at day 21 after the labeling and perfused with 4% paraformaldehyde in phosphate buffered saline (PBS).

### Immunohistochemistry and neutral red staining

Mice were sacrificed and perfused with 4% paraformaldehyde in 0.1 M phosphate buffer (PB, pH 7.4). The brain, spinal cord or sciatic nerve was dissected out, immersion-fixed in the same fixative for 6 h, and then placed in 30% sucrose in 0.1 M PBS overnight and were cut into 30 *μ*m-thick sections with a cryostat (Leica, Ontario, Canada). Spinal cord sections were stained with 1% Neutral red as described previously [45, 46].

For immunohistochemistry, sections of the brain, spinal cord or sciatic nerves were treated according to standard procedures [23, 24]. Briefly, the sections were incubated overnight at room temperature with the primary antibodies. Table 1 shows the names of the antibodies, vendors and concentrations used in this study. After rinsing with 0.01M PBS, they were incubated for 1 h at room temperature with corresponding secondary antibodies conjugated with Alexa-488 or 568 (Molecular Probes, Eugene, USA). The primary and secondary antibodies were diluted in PBS containing 1% normal goat serum and 0.2% Triton X-100. Each step was followed by three washes in the PBS. At last, the sections on gelatin-coated glass slides were coverslipped in mounting medium (Dako, Denmark). Fluorescent images were captured with a Zeiss microscope (Zeiss, Gottingen, Germany) equipped with a Spot digital camera (Diagnostic Instruments, Sterling Heights, MI, USA). 10 sections for each animal were examined.

**Table 1:** Antibodies used in the experiments

Antibodies	Species	Working dilution	Vendor or producer
ChAT	Goat IgG	1:800	Chemicon
Aβ40	Rabbit IgG	1:2000	Chemicon
Bam-10	Mouse IgG	1:4000	Sigma
NF200	Rabbit IgG	1:1000	Sigma
APP	Rabbit IgG	1:1000	Sigma

### Bielshowsky silver staining

Axonopathy was further assessed by Bielshowsky silver staining according to previous studies [29]. Briefly, staining, sections were placed for 20 min in Silver Solution 1 (10% AgNO_3_ in H_2_O) then rinsed twice in water and incubated in Silver Solution 2 (200 ml of Silver Solution 1 plus NH_3_) for 15 min in the dark. After these steps, the sections were washed in 200 ml of water plus 5 drops of NH3 and put in Silver Solution 3 (Silver Solution 2 plus 500 ul of development solution made of 4% formaldehyde, 0.5 g of monohydrate citric acid and 1–2 drops of concentrated HNO_3_) to obtain the optimal staining. The sections were then rinsed twice in water and then in 5% natrium thiosulfate in H_2_O, dehydrated through increasing concentrations of ethanol, put in n-butyl acetate and assembled.

### Tissue preparation for electronic microscopy (EM)

Tissue preparation for EM followed the procedure described in our previous study [47]. Briefly, animals were euthanized with an overdose of Dorminal 20% (each ml contains 200 mg pentobarbital sodium, Alfasan, Woerden-Holland), then transcardially perfused with PBS, pH 7.4, containing 2% paraformaldehyde and 2.5% glutaraldehyde. Spinal cord in cervical segments was carefully dissected and 1mm-thick cross sections were cut and placed in the same fixatives overnight at 4°C. Sections were further post-fixed in 2% osmium tetroxide, dehydrated through a graded acetone series, and embedded in EPON. Ultrathin cross and horizontal sections were stained with 3% uranyl acetate and 1% lead citrate. Images for axon morphology were examined and captured by transmission electron microscopy (TEM, Phillip model 208).

### Statistical analysis

Data from TgCRND8 and non-TgCRND8 animals were presented as mean ± SEM. Differences were tested using Student's *t*-test. Statistical significance was set at *p* < 0.05.
